# Bioreactance and fourth-generation pulse contour methods in monitoring cardiac index during off-pump coronary artery bypass surgery

**DOI:** 10.1007/s10877-021-00721-0

**Published:** 2021-05-26

**Authors:** Laura Anneli Ylikauma, Pasi Petteri Ohtonen, Tiina Maria Erkinaro, Merja Annika Vakkala, Janne Henrik Liisanantti, Jari Uolevi Satta, Tatu Sakari Juvonen, Timo Ilari Kaakinen

**Affiliations:** 1 Medical Research Center Oulu, Research Group of Surgery, Anesthesiology and Intensive Care Medicine, Oulu University Hospital and University of Oulu, Oulu, Finland; 2 Division of Operative Care, Oulu University Hospital, Oulu, Finland; 3 Department of Cardiac Surgery, Heart and Lung Center, Helsinki University Central Hospital, Helsinki University Hospital, Helsinki, Finland

**Keywords:** Bioreactance, Cardiac index and output, Mini-invasive, Monitoring, Non-invasive, Pulse contour

## Abstract

The pulmonary artery catheter (PAC) is considered the gold standard for cardiac index monitoring. Recently new and less invasive methods to assess cardiac performance have been developed. The aim of our study was to assess the reliability of a non-invasive monitor utilizing bioreactance (Starling SV) and a non-calibrated mini-invasive pulse contour device (FloTrac/EV1000, fourth-generation software) compared to bolus thermodilution technique with PAC (TDCO) during off-pump coronary artery bypass surgery (OPCAB). In this prospective study, 579 simultaneous intra- and postoperative cardiac index measurements obtained with Starling SV, FloTrac/EV1000 and TDCO were compared in 20 patients undergoing OPCAB. The agreement of data was investigated by Bland–Altman plots, while trending ability was assessed by four-quadrant plots with error grids. In comparison with TDCO, Starling SV was associated with a bias of 0.13 L min^−1^ m^−2^ (95% confidence interval, 95% CI, 0.07 to 0.18), wide limits of agreement (LOA, − 1.23 to 1.51 L min^−1^ m^−2^), a percentage error (PE) of 60.7%, and poor trending ability. In comparison with TDCO, FloTrac was associated with a bias of 0.01 L min^−1^ m^−2^ (95% CI − 0.05 to 0.06), wide LOA (− 1.27 to 1.29 L min^−1^ m^−2^), a PE of 56.8% and poor trending ability. Both Starling SV and fourth-generation FloTrac showed acceptable mean bias but imprecision due to wide LOA and high PE, and poor trending ability. These findings indicate limited reliability in monitoring cardiac index in patients undergoing OPCAB.

## Introduction

Mortality related to cardiac surgery has decreased in recent decades [1]. Still there is a need to improve the survival rate among cardiac surgery patients. Goal-directed therapy (GDT) using fluids, vasopressors and inotropes has been shown to reduce postoperative complications and to shorten the length of hospital stay after cardiac surgery [1–3]. GDT is implemented to optimize oxygen delivery, although the targets, monitoring methods and subsequent therapy interventions are still heterogeneous [1, 2]. Simple hemodynamic measurements such as heart rate and mean arterial pressure (MAP) alone are not reliable in detecting reduced oxygen delivery [4]. Oxygen delivery can be improved by measuring and optimizing cardiac output (CO) [5]. There are various CO monitors available, and continuous CO monitors and their ability to detect changes in CO have recently gained interest [6]. The CO monitors used to guide GDT need to be reliable in measuring CO, otherwise the conclusions cannot be adapted to clinical use [1].

The pulmonary artery catheter (PAC) has been the gold standard for CO monitoring since the 1970s [7–9]. PAC is a highly invasive intermittent CO monitor which is associated with a risk of serious complications [9, 10]. Less invasive and continuous hemodynamic monitors have been developed, and there are numerous different devices commercially available. A totally non-invasive CO monitor, Starling SV, is based on a transthoracic bioreactance technique allowing continuous measurement of CO [11]. The mini-invasive non-calibrated FloTrac/EV1000 uses arterial pressure waveform analysis to calculate SV and thereby CO [12]. Successful CO monitoring relies on the accuracy and precision of the measurements and the ability to detect short term changes in CO values [5]. The accuracy and precision of the less invasive hemodynamic monitors have not yet been shown to be interchangeable with the bolus thermodilution technique with a PAC (TDCO) [5, 8, 13–15]. Studies on the subject often have limitations such as insufficient number of samples, limited statistical methods, failure to use an accepted reference technique or present no assessment of trending ability [6].

Our hypothesis was that non-invasive bioreactance method (Starling SV), non-calibrated mini-invasive pulse contour method (FloTrac) and TDCO are equally reliable when monitoring patients undergoing off-pump coronary artery bypass surgery (OPCAB). The aim of the present study was to assess the agreement in accuracy, precision and trending ability of Starling SV and FloTrac compared to TDCO in the setting of OPCAB.

## Methods

This prospective single-center observational method comparison study was approved by the Ethics Committee of Oulu University Hospital (66/2017, 14/08/2017). The patients were properly informed both orally and in writing before obtaining the study consent. We included 20 consecutive patients undergoing OPCAB surgery between March and June 2018, and our exclusion criterion was the refusal of the patient to attend the study. Since the present study is only a monitoring method comparison study, therapeutic decisions were based on TDCO measurements according to local clinical practice.

The patients were anesthetized with intravenous infusions of propofol and remifentanil, and rocuronium was administered to achieve neuromuscular blockade. General anesthesia was maintained with a combination of sevoflurane and propofol, and intraoperative analgesia was provided with remifentanil. Postoperatively, the patients were transferred to the intensive care unit (ICU) with the propofol and remifentanil infusions. In the ICU, remifentanil was replaced with intravenous oxycodone (bolus or infusion). Patients were awakened and extubated according to local fast-track principles.

Upon arrival in the operation theatre (OR), a 7.5F PAC (Criticath SP5507U TD Catheter, Merit Medical, South Jordan, Utah, USA) was inserted via an 8.5F sheath placed in the right internal jugular vein and advanced into the pulmonary artery until a wedge pressure trace was obtained. TDCO measurements were obtained with bolus injections of 10 ml 20 °C 0.9% saline at room temperature and cardiac index (CI) was determined as an average of at least three measurements [16]. The measurements were not synchronized with the respiratory cycle [17].

The bioreactance-based non-invasive Starling SV (CMM-ST5, 2017-12-01, Version 5.2, Cheetah Medical, Newton, Massachusetts, USA) produces an alternating electrical current through the thorax. The pulsatile blood flow taking place in the large thoracic arteries creates time delays (or phase shifts) between the applied current and the measured thoracic voltage. As the bioreactance signal is almost entirely correlated with aortic flow, there is a proportional relationship between the phase shifts and cardiac output. [11, 13, 18, 19] Starling SV consists of four dual-electrode stickers that were placed on the back of the patients, two of them on the right and two of them on the left side of the chest wall according to the instructions [19]. The backside was selected due to the cardiac surgery setting. Starling SV calibrates itself automatically at the start of the monitoring session and can be recalibrated as needed. In practice, it was recalibrated when the position of the patient or the heart was significantly altered, e.g. when the heart was lifted upwards to suture the distal coronary anastomoses, or if the signal appeared to be unreliable.

Mini-invasive FloTrac (Version 4.00, Edwards Lifesciences, Irvine, California, USA) is based on the principle that aortic pulse pressure is proportional to stroke volume, and its undisclosed algorithm uses the MAP, arterial pressure waveform analysis and arterial compliance to calculate SV and thereby CO [11–13]. Waveform characteristics assessed are skewness and kurtosis, which reflect changes in vascular tone [11]. The patient’s vascular compliance is assessed using patient data (age, sex, weight and height). FloTrac cannot be calibrated [11, 13, 14]. In our study, FloTrac was connected to an arterial line placed into the radial or brachial artery (BD Arterial Cannula 20G, Becton Dickinson and Company, Franklin Lakes, New Jersey, USA).

We performed 579 simultaneous measurements of CI which were taken both intraoperatively in the OR and postoperatively in the ICU until the first postoperative morning. In the OR, the measurements were taken every time the position of the heart was significantly changed or it was otherwise necessary to guide the treatment of the patients, or at least every 30 min. In the ICU the measurements were taken approximately once in an hour in the evening and every 2–3 h in the night-time. The sample size was calculated post hoc for an equivalence study [20]. We used data from the present study, in which the mean CI of TDCO is 2.4 and the mean CI of Starling SV is 2.2. The results were: standard deviation of differences (SD) 0.7, non-inferiority margin 0.36, alpha 0.05, beta 0.10 (power 0.9), giving a sample size of 414 measurements.

Both the OR and the ICU have advanced electronic medical record systems, which we used to obtain continuous hemodynamic data from TDCO and FloTrac. The data from Starling SV was recorded continuously into its own database, and we collected this data afterwards using distinct hemodynamic changes as landmarks. The patient monitor we used was Carescape B850 Monitor (GE Healthcare, Chicago, Illinois, USA).

In addition to analyzing all the measurements, we specifically concentrated on distinct phases that are hemodynamically the most challenging in the OR or ICU. Therefore, we separately present the results of the measurements which were taken just before the induction of anesthesia (phase 1, baseline), during side-clamping of the aorta when the proximal anastomoses to the ascending aorta are constructed (phase 2), during the distal coronary anastomoses when the heart is in a vertical position (phase 3), and those taken in the ICU before extubation (phase 4).

### Statistics

CI was used for the analysis instead of CO as it is used in clinical settings in our hospital. Our summary statistics are presented as medians with 25th–75th percentiles [25–75 PCT] unless stated otherwise. Two-tailed p values are presented. The mean bias between measurements and limits of agreement (LOA) with 95% confidence intervals (95% CI) were calculated according to Bland and Altman [6, 21–26] The data structure with multiple independent measurements within the subject was taken into account while calculating LOA, using the method where the true value varies [22, 24]. We also calculated regression coefficients with 95% CI to evaluate proportional bias to assess whether the difference between the techniques varies depending on the magnitude of the CI, thereby skewing the bias. Since the bias and LOA are uniform in our study, we report the regression coefficients as absolute values [6]. The percentage errors (PE) with 95% CI were calculated for each phase [6]. Furthermore, to assess the trending ability of the study monitors, four-quadrant (4Q) plots consisting of the changes of two consecutive CI measurements were constructed with exclusion zones as recommended in the literature. Based on the clinical concordance categories of the 4Q plot, error grids were created to generate four zones to define the level of agreement between changes in CI measured by two devices. In zone 1 the CI has changed in the same direction to the same extent or in other words, both have changed less than 5%, between 5–15%, or over 15%, leading to uniform treatment decisions. In zone 2 the CI has changed in the same direction but not to the same extent, reflecting insufficient or exaggerated treatment. In zone 3 only one of the measured CI values has changed, implying that unnecessary treatment may be initiated or, or necessary treatment withheld. In zone 4 the changes have been opposite, and opposite treatment may be initiated [6]. Analyses were performed using SPSS for Windows (IBM Corp. IBM SPSS Statistics for Windows, Version 25.0. Armonk, NY: IBM Corp.) and SAS for Windows (version 9.4 SAS Institute Inc., Cary, NC, USA).

## Results

The patient characteristics are presented in Table 1. The median age of the patients was 68 years and 90% of them were male. Half of the patients underwent urgent OPCAB surgery during hospitalization due to acute coronary syndrome, while the rest were elective cases. There was no hospital mortality. The median of measurements per patient was 30. 559 delta CI measurements were used in the 4Q plot.

**Table 1:** Patient characteristics (n = 20)

Age, years	68 (64–70)
Sex male	18 (90)
Weight, kg	84 (69–100)
BSA, m^2^	1.98 (1.82–2.16)
BMI, kg m^−2^	28 (24–33)
Prior co-morbidities	
Hypertension	14 (70)
Type 2 diabetes mellitus	9 (45)
COPD	3 (15)
Asthma	2 (10)
Left ventricular hypertrophy	7 (35)
Atrial fibrillation	4 (20)
Medication prior to surgery	
Acetylsalicylic acid	17 (85)
Clopidogrel	1 (5)
Low molecular weight heparin	7 (35)
Beta blocker	15 (75)
Statin	16 (80)
ACE inhibitor or AT II receptor inhibitor	12 (60)
Long-acting nitrate	8 (40)
Medical state prior to surgery	
Ejection fraction	
> 50%	16 (80)
31–50%	2 (10)
21–30%	2 (10)
Coronary artery stenoses	
RCA	20 (100)
CX	17 (85)
LAD	19 (95)
LM	7 (35)
NYHA class	3 (2–3)
Euroscore II, %	1.43 (0.90–2.27)
Hemoglobin, g L^−1^	141 (130–157)
Thrombocytes, E9 L^−1^	255 (208–307)
INR	1.0 (1.0–1.1)
Surgery	
Urgency	
Urgent	10 (50)
Elective	10 (50)
Number of bypasses	4 (3–4)
Levosimendan used	5 (25)
Norepinephrine max dose, microg kg^−1^ min^−1^	0.18 (0.12–0.44)
Dobutamine max dose, microg kg^−1^ min^−1^	2.00 (0.00–2.74)
I.v. nitrate used	14 (70)
OR stay, min	404 (344–440)
Time in ventilator, OR and ICU combined, h	9 (8–12)
ICU length of stay, days	1 (1–3)
Hospital length of stay, days	9 (8–13)
Hospital mortality	0 (0)

The values given are medians with 25th and 75th percentiles, or number of patients (n) with percentages (%). *BSA* body surface area, *BMI* body mass index, *RCA* right coronary artery, *CX* circumflex artery, *LAD* left anterior descending artery, *LM* left main artery, *NYHA Class* New York Heart Association Classification, *INR* international normalized ratio, *OR* operating theatre, *ICU* intensive care unit

**Fig. 1:**
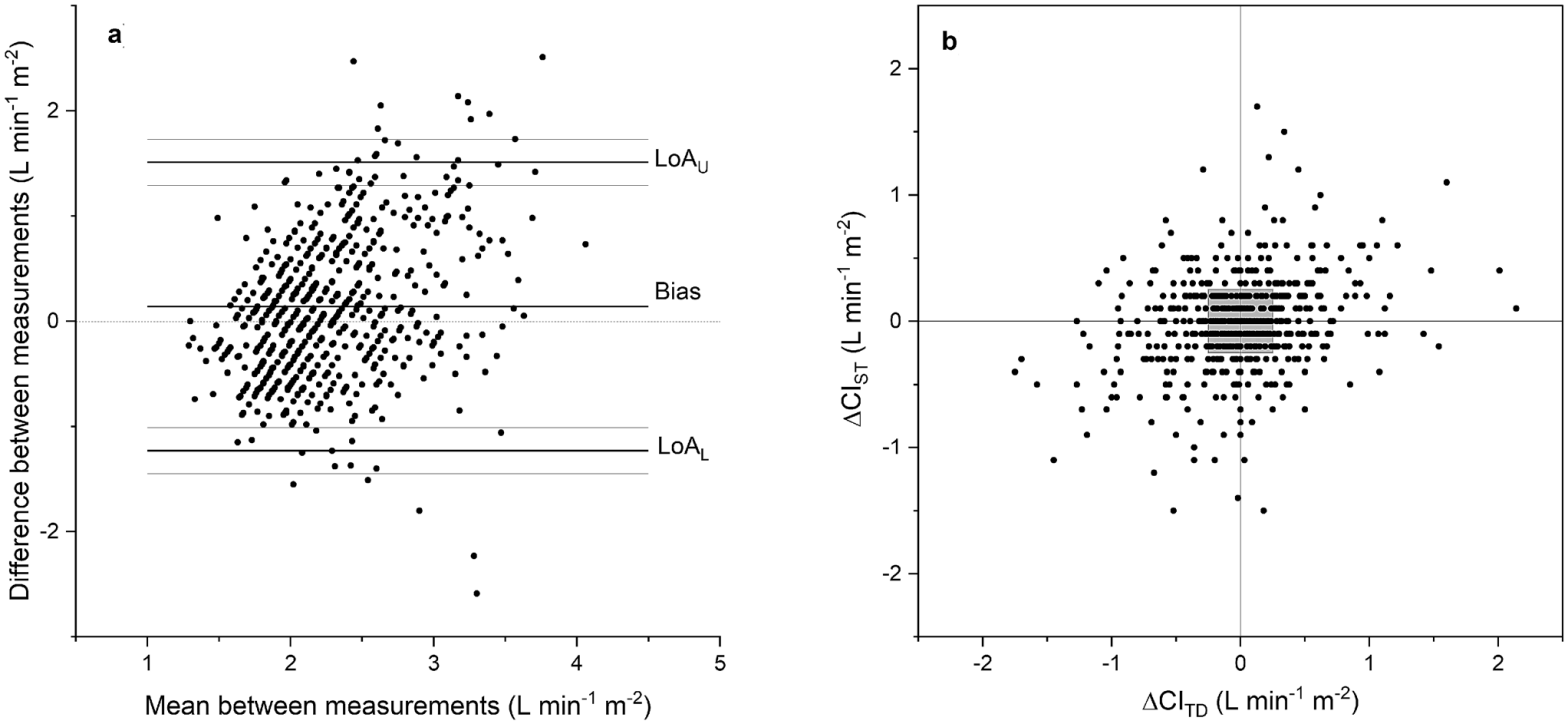
**a** The Bland–Altman plot for cardiac index determined by the bolus thermodilution technique with a pulmonary artery catheter and bioreactance-based Starling SV, all measurement points. The lines for bias, LOA and 95% CIs of LOA are shown. See also Table 2 for exact numbers. **b** The 4-quadrant method plots the change of consecutive CI measured with Starling SV (ΔCI_ST_) against the change in our reference method thermodilution (ΔCI_TD_) showing the trending ability of Starling SV at all the measurement points. See also Table 2 for exact numbers

In comparison with TDCO, considering all measurements over the study protocol, Starling SV was associated with a bias of 0.13 L min^−1^ m^−2^ (95% CI 0.07 to 0.18) and LOA of − 1.23 to 1.51 L min^−1^ m^−2^ (Fig. 1a). Figure 1b shows the 4Q method plotting the changes in CI measured by Starling SV against the changes in CI measured by TDCO. In the error grid based on the 4Q plot, the level of agreement in trending was 29.0% in zone 1. The results between Starling SV and TDCO are presented in Table 2.

**Table 2:** Cardiac index measurements by Starling SV compared to bolus thermodilution technique with a pulmonary artery catheter

Starling SV	All n = 579	Phase 1 n = 24	Phase 2 n = 27	Phase 3 n = 85	Phase 4 n = 104
Bias (L min^−1^ m^−2^) (95% CI)	0.13 (0.07 to 0.18)	0.21 (− 0.04 to 0.46)	0.21 (− 0.07 to 0.49)	− 0.36 (− 0.49 to − 0.24)	− 0.02 (− 0.16 to 0.12)
LOA lower (L min^−1^ m^−2^) (95% CI)	− 1.23 (− 1.45 to − 1.01)	− 0.83 (− 1.26 to − 0.41)	− 1.08 (− 1.61 to − 0.56)	− 1.45 (− 1.82 to − 1.08)	− 1.34 (− 1.79 to − 0.9)
LOA upper (L min^−1^ m^−2^) (95% CI)	1.51 (1.29 to 1.73)	1.38 (0.95 to 1.8)	1.57 (1.05–2.1)	0.92 (0.54 to 1.29)	1.44 (1.0 to 1.88)
Percentage error (95% CI)	60.7% (51.5 to 69.9)	46.0% (31.3 to 60.7)	60.0% (42.9 to 77.1)	53.2% (32.1 to 74.3)	65.6% (43.6 to 87.6)
Regression coefficient (L min^−1^ m^−2^) (95% CI)	0.34 (0.21 to 0.47)	0.39 (− 0.02 to 0.79)	0.31 (− 0.31 to 0.93)	0.28 (− 0.09 to 0.64)	0.09 (− 0.26 to 0.43)
Error grid					
Zone 1	29.0%	–	–	28.4%	26.0%
Zone 2	15.3%	–	–	18.5%	15.4%
Zone 3	34.3%	–	–	37.0%	30.8%
Zone 4	21.4%	–	–	16.0%	27.9%

Phase 1 is before induction of anesthesia, phase 2 is during the construction of the proximal anastomoses to the ascending aorta, phase 3 is distal coronary anastomoses, phase 4 is postoperatively in the intensive care unit before extubation. Error grid analysis was not made for the phases 1 and 2 because of the small number of measurements. n number of CI measurements during the phase. *LOA* limits of agreement

**Fig. 2:**
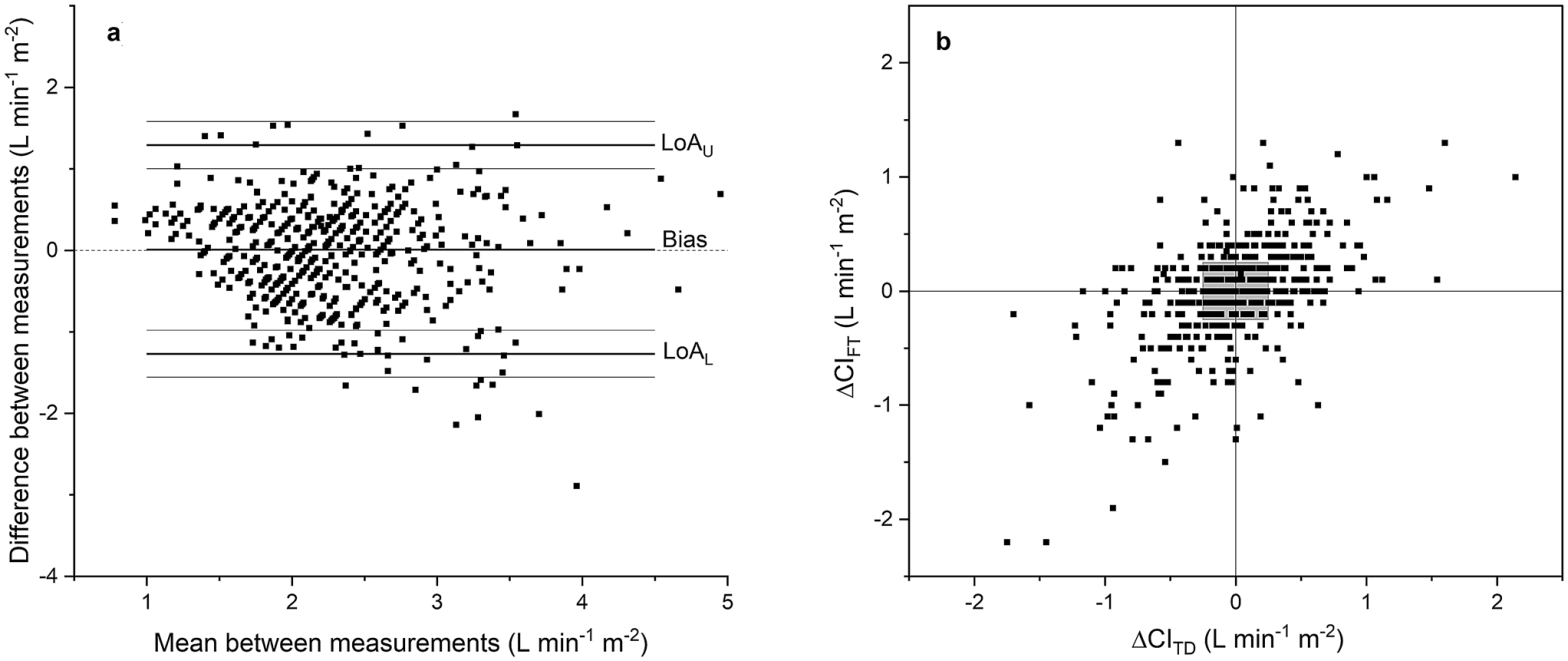
**a** The Bland–Altman plot for cardiac index determined by the bolus thermodilution technique with a pulmonary artery catheter and pulse contour system FloTrac, all measurement points. The lines for bias, LOA and 95% CIs of LOA are shown. See also Table 3 for exact numbers. **b** The 4-quadrant method plots the change of consecutive CI measured with FloTrac (ΔCI_FT_) against the change in our reference method thermodilution (ΔCI_TD_) showing the trending ability of FloTrac at all the measurement points. See also Table 3 for exact numbers

In comparison with TDCO, considering all measurements over the study protocol, FloTrac was associated with a bias of 0.01 L min^−1^ m^−2^ (95% CI − 0.05 to 0.06) and LOA of − 1.27 to 1.29 L min^−1^ m^−2^ (Fig. 2a). Figure 2b shows the 4Q method plotting the changes in CI measured by FloTrac against the changes in CI measured by TDCO. In the error grid based on the 4Q plot, the level of agreement in trending was 39.3% in zone 1. The results between FloTrac and TDCO are presented in Table 3.

**Table 3:** Cardiac index measurements by FloTrac compared to bolus thermodilution technique with a pulmonary artery catheter

FloTrac	All n = 579	Phase 1 n = 24	Phase 2 n = 27	Phase 3 n = 85	Phase 4 n = 104
Bias (L min^−1^ m^−2^) (95% CI)	0.01 (− 0.05 to 0.06)	− 0.17 (− 0.44 to 0.10)	− 0.01 (− 0.27 to 0.26)	− 0.15 (− 0.30 to 0.00)	− 0.25 (− 0.39 to − 0.11)
LOA lower (L min^−1^ m^−2^) (95% CI)	− 1.27 (− 1.56 to − 0.98)	− 1.28 (− 1.8 to − 0.77)	− 1.32 (− 1.85 to − 0.78)	− 1.52 (− 1.99 to − 1.06)	− 1.78 (− 2.37 to − 1.18)
LOA upper (L min^−1^ m^−2^) (95% CI)	1.29 (1.0 to 1.58)	0.93 (0.41 to 1.45)	1.26 (0.72 to 1.79)	1.08 (0.61 to 1.54)	1.27 (0.67 to 1.87)
Percentage error (95%CI)	56.8% (44.4 to 69.2)	47.2% (32.8 to 61.6)	53.7% (32.7 to 74.7)	65.7% (48.1 to 83.3)	61.9% (30.4 to 93.4)
Regression coefficient (L min^−1^ m^−2^) (95% CI)	− 0.19 (− 0.27 to − 0.10)	− 0.12 (− 0.67 to 0.43)	− 0.26 (− 0.98 to 0.47)	− 0.06 (− 0.37 to 0.26)	− 0.31 (− 0.52 to − 0.09)
Error grid					
Zone 1	39.3%	–	–	36.8%	45.5%
Zone 2	12.2%	–	–	11.8%	8.0%
Zone 3	35.4%	–	–	31.6%	38.6%
Zone 4	13.0%	–	–	19.7%	8.0%

Phase 1 is before induction of anesthesia, phase 2 is during the construction of the proximal anastomoses to the ascending aorta, phase 3 is distal coronary anastomoses, phase 4 is postoperatively in the intensive care unit before extubation. Error grid analysis was not made for the phases 1 and 2 because of the small number of measurements. n number of CI measurements during the phase. *LOA* limits of agreement

## Discussion

In this study, we compared the non-invasive Starling SV and the mini-invasive fourth-generation FloTrac to TDCO in measuring the cardiac index in patients undergoing OPCAB surgery. The main results are that both two monitors show acceptable mean bias but are not precise enough due to wide LOA and high PE, and their trending abilities are poor. These findings indicate the limited reliability of both Starling SV and fourth-generation FloTrac in the setting of OPCAB surgery.

When investigating new CO monitors, a reliable reference device is needed. Choosing an inaccurate and imprecise reference device results in rejecting the new device. [27] We chose TDCO as our reference method. PAC is considered the gold standard for CO monitoring [7–9]. The precision of TDCO has been studied widely and proved in the literature to be  ± 20% at the most and thus it is considered acceptable as a reference method for measuring CO [6, 28–33] Testing TDCO in vitro also resulted in good precision [34]. Despite its associated risk of serious complications [10], PAC still has an important role in monitoring and treating critically ill patients [35]. PAC is also a part of our standard clinical protocol in cardiac surgery patients.

We compared both Starling SV and FloTrac independently to our reference method TDCO. The Bland–Altman analysis evaluates the agreement between two CO measurement techniques. It determines the bias as a measure of accuracy while the 95% LOA describes precision. [6, 21]. It is a clinical decision which levels of agreement between a new and a reference CO measurement technique are acceptable. It has been suggested that a bias of 0.5 L min^−1^ and LOA of  ± 1.0 L min^−1^ are acceptable when measuring CO in patients undergoing surgery with major hemodynamic disturbances [6], and PE as a sign of precision should not exceed 30% [30]. Based on these values the accuracy of both our study monitors was acceptable while precision was inadequate. Although there is some evidence on proportional bias in terms of the significant regression coefficient (Tables 2, 3), the regression coefficients in our study are relatively small (0.39 L min^−1^ m^−2^ at most with Starling SV and − 0.31 L min^−1^ m^−2^ with FloTrac). However, some spread of the bias with higher CI values can be seen in the Bland–Altman figures (Figs. 1a, 2a).

There are only a few studies evaluating the accuracy and precision of Starling SV in cardiac surgery patients. A good accuracy compared with continuous thermodilution with a PAC (PAC-CCO) was found with an earlier version of the bioreactance technique (NICOM) during OPCAB surgery, but the LOA were not reported and PE was not calculated [36]. Comparable accuracy and precision between NICOM and PAC-CCO was reported in patients after cardiac surgery [37]. But as the PAC-CCO is not a valid reference technique the results are not comparable to ours [6, 32, 37, 38]. In a study employing several non-surgical settings, similar results to ours were reported with acceptable bias but wide LOA between NICOM and TDCO [19], whereas another study comparing bioreactance to transpulmonary thermodilution showed inaccuracy as well as imprecision [6, 39].

There have been improvements in the monitor software during recent years with the FloTrac device, as previous generations of FloTrac have shown poor agreement compared with TDCO [11, 31, 38, 40–43]. A common assumption has been that abnormal systemic vascular resistance (SVR) reduces the reliability of pulse contour analysis [11, 31, 41]. The fourth-generation software was created to overcome the problems with earlier generations of FloTrac [44]. However, only a few previous studies compare the fourth-generation FloTrac to TDCO. In accordance with our results, a study on OPCAB surgery found a good accuracy but imprecision, as the bias was acceptable − 0.05 L min^−1^ but LOA were wide (− 1.47 to 1.37 L min^−1^), PE was 33.8%, and the trending ability was poor [28]. A study comparing the fourth-generation FloTrac to TDCO during cardiac surgery with pulmonary bypass reported inaccuracy, imprecision and poor trending ability [33]. Similar results were found when comparing FloTrac to PAC-CCO [44].

When assessing the reliability and clinical use of a CO device, in addition to its accuracy and precision, it is also crucial to evaluate its ability to track changes reliably [5]. We assessed trending ability with the 4Q method, which plots the change of CO in the experimental device against the change of CO in the reference method (Figs. 1b, 2b) [6, 45]. The error grid uses four zones to define the level of agreement between changes in CO measured by two devices. In zone 1 the CO has changed in the same direction to the same extent, whereas in zone 4 the changes have been opposite [6]. Our results showed poor trending ability since only 29.0% and 39.3% of the data points were on in zone 1 with Starling SV and FloTrac, respectively. 21.4% and 13.0% of the data points of Starling SV and FloTrac, respectively, were in zone 4.

In OPCAB surgery, there are various significant hemodynamic challenges related to the surgical method, which indicates that careful perioperative hemodynamic monitoring is required. Low SVR, myocardial ischemia caused by coronary occlusion during construction of the distal anastomoses, the mobilization and stabilization of the heart causing heart dislocation, the compression of the right ventricle, the compression of the left ventricular outflow tract, mitral and tricuspid insufficiency during abnormal cardiac position and abnormal diastolic expansion can be specific challenges in OPCAB surgery. These challenges can also affect the reliability of CO monitoring perioperatively [46, 47]. Especially low SVR is reported to reduce the reliability of FloTrac [11, 31, 41]. During side-clamping of the aorta the aortic impedance changes [48], which may affect the CO measurements by both Starling SV and FloTrac. In addition, TDCO underestimates the actual CO during tricuspid regurgitation [49]. In the present study, aortic side-clamping (phase 2) or vertical positioning of the heart (phase 3) did not have any impact on our results. However, these situations are not optimal for reliable TDCO measurements, but are hemodynamically challenging in a clinical setting.

Our study has several strengths. We used the gold standard TDCO as our reference method. The study setting was a prospective case series, and we used statistical methods that are recommended in studies comparing different methods of cardiac output monitoring [5]. As our medical record systems are electronic, we collected the data continuously and reliably. A weakness of our study is that we calculated our sample size post hoc. However, we used the method recommended in the literature [20] and considered the data structure with multiple independent measurements within the subject, as we had 20 patients but 579 simultaneous measurements. Moreover, we also assessed the sample size post hoc according to Bland et al. with an expected 95% CI of LOA being 0.2 L min^−1^ m^−2^, producing a sample size of at least 141 samples [21, 50].

It is also a weakness of our study that we did not determine the precision of TDCO. However, in the literature the precision of TDCO has been proven to be at most  ± 20% [6, 28–33]. We obeyed the recommendation to perform at least 3 measurements when measuring CI with PAC, and each time the thermodilution curve was carefully checked and unreliable curves were erased [16]. Thus, the possible variation in TDCO precision was limited. We did not define acceptable bias and LOA in advance as recommended in the literature [6], but the conclusions of our study would not have changed even if we had done so. Our reference technique was intermittent and the experimental devices are continuous cardiac output monitors, so there is a difference in response time during hemodynamic changes. This can be seen as a limitation of our study. In the acute setting though, the therapeutic decisions need to be done fast based on the monitor information, and the response times of the monitors should be short [6].

Our patients median Euroscore II (estimated risk of in-hospital death after cardiac surgery) was 1.43%, and 80% of our patients had ejection fraction more than 50%, which may limit the applicability of our results to more diseased patients. However, as the performance of our experimental monitors was unsatisfactory even under these relatively stable conditions, it is unlikely that they would yield more reliable information during hemodynamic challenges. High-risk cardiac surgery is not the ideal setting to test the reliability of the non- and mini-invasive CI monitors, considering their limitations. Yet, as PAC is invasive and its use is essential to guarantee a high quality study setting, newer CI monitors need to be studied under surgical settings where the use of TDCO is ethical, safe and clinically reasonable.

In conclusion, both Starling SV and fourth-generation FloTrac showed acceptable accuracy, but imprecision due to wide LOA and high PE, and poor trending ability indicating limited reliability in monitoring cardiac index in patients undergoing OPCAB surgery. Our hypothesis about equal reliability of the monitors was not supported by the results.

## Data Availability

The data that support the findings of this study are available from the corresponding author, LY, upon reasonable request.
